# Dietary Patterns, Socio-Demographic Predictors Thereof, and Associations of Dietary Patterns with Stunting and Overweight/Obesity in 1–<10-Year-Old Children in Two Economically Active Provinces in South Africa

**DOI:** 10.3390/nu15194136

**Published:** 2023-09-25

**Authors:** Marjanne Senekal, Johanna H. Nel, Gabriel Eksteen, Nelia P. Steyn

**Affiliations:** 1 Department of Human Biology, University of Cape Town, Cape Town 7925, South Africa; nelia.steyn@uct.ac.za; 2 Department of Logistics, Stellenbosch University, Stellenbosch 7602, South Africa; jhnel@sun.ac.za; 3 Clinical and Experimental Endocrinology, Department of Chronic Diseases and Metabolism, KU Leuven, 3000 Leuven, Belgium; gabrieljohannes.eksteen@kuleuven.be

**Keywords:** dietary patterns, stunting, overweight, obesity, children < 10 years, South Africa

## Abstract

A review of the literature showed that there were only a few studies that reported on the dietary patterns of children in South Africa. The aim of the present study was to characterise the dietary patterns of children aged 1–<10 years who were studied during the Provincial Dietary Intake Survey (PDIS) in 2018 and to investigate the socio-demographic predictors thereof, as well as the associations with stunting and overweight/obesity. Dietary pattern analysis was conducted within three age groups, namely 1–<3-year-olds, 3–<6-year-olds, and 6–<10-year-olds using iterated principal factor analysis with varimax rotation and 24 h recall data from the PDIS. The dietary patterns that emerged seem to be far from ideal. Energy-dense, nutrient-poor patterns were included in the top three strongest patterns in all three age groupings that were investigated. Few of the dietary patterns included vegetables other than starchy vegetables, fruit, dairy, quality proteins, and unrefined carbohydrates. There were no associations between any of the dietary patterns and stunting or overweight/obesity in the children. Key predictors of greater adherence to the mostly unhealthy patterns included indicators of a higher socio-economic status in all three age groups, as well as having an obese mother in the 6–<10-year-old group. Key predictors of greater adherence to the mostly healthy patterns were a higher wealth index and having an obese mother in the two younger groups, with no predictors in the 6–<10-year-old group. We conclude that the dietary patterns of children in the Western Cape contain strong elements of the energy-dense, nutrient-poor dietary patterns. Interventions to improve the dietary intake of children should be directed at both poorer and higher income communities.

## 1. Introduction

The Joint Malnutrition Estimates (JME) report that, globally, 149.2 million children under five years of age are stunted, 45.4 million are wasted, and 38.9 million are overweight. Overweight and obesity have emerged as global challenges, affecting low- and high-income countries alike. Their figures show that the share of both adults and children who are overweight or obese is increasing globally [1].

Optimal nutrition during infancy and childhood is essential for the growth and development of children. Malnutrition in children can result in a range of nutrition-related health problems, depending on the nature thereof, i.e., deficient and/or excessive intakes of energy, macro, and micronutrients. Childhood malnutrition phenotypes that are a concern in South Africa include stunting, overweight/obesity, iron anaemia (iron deficiency), and low vitamin A levels [2].

Malnutrition during childhood has been linked to poor cognitive development and, over time, the promotion of the development of non-communicable disease [3]. According to Liberali et al. [4], one of the most serious public health problems of the 21st century in both low-, middle-, and high-income countries is the fact that childhood obesity is a predictor of risk of obesity in adulthood. Not only is childhood obesity associated with adult obesity but also with increased risk for numerous non-communicable diseases (NCDs) such as diabetes, cardiovascular diseases (CVDs), and certain cancers [5,6].

In South Africa, as in many sub-Saharan African countries, a double burden of malnutrition is prevalent in children [2]. A recent study of children under five in two provinces in South Africa clearly illustrates the prevalence of the double burden of malnutrition in children per se. The prevalence of stunting in under five-year-olds was 21.6%, underweight 5.6%, overweight 10.3%, and obesity 7.0%. In the five- to younger than 10-year-old group, 6.7% were stunted, 6.8% underweight, 13.4% overweight, and 6.8% obese [7]. A comparison of anthropometric indicators in 1–9-year-olds in the Western Cape (WC) province between 1999 and 2018 shows no increase in stunting in the total group of children (14.9–13.7%), but an increase in overweight/obesity (13.5–21.8%) [8]. The 2022 Obesity Atlas projects that 28.2% of 5–9-year-old children in South Africa will be obese by 2030 [9].

While many NCDs may not present until later in life, dietary habits and patterns are generally formed in childhood and continue into adulthood [10]. A World Health Organization (WHO) systematic review concluded that a person’s predisposition to developing obesity and other NCDs can be influenced as early as during foetal development and during childhood [11]. This is confirmed by researchers exploring what is known as the “life course of disease approach” to NCD prevention and control. This approach contends that all areas of life should be considered in relation to controlling NCD rates from preconception and prenatal care, to infancy, childhood, adolescence, adulthood, and the elderly [12]. The “life course of disease approach” thus advocates for the importance of educating and introducing intervention strategies from early in life.

The Global Burden of Disease Study (GBDS) noted that the highest rates of mortality and disability-adjusted life years (DALYs) related to diet were recorded in low- and middle-income countries [13]. The risk factors associated with the highest rate of mortality in these countries descending order were as follows: “a diet high in sodium, low in whole grains, low in fruits, low in nuts and seeds, low in vegetables, low in fish/seafood (omega-3-rich fatty acids), low in fibre, low in poly-unsaturated fats, low in legumes, high in trans fats, low in calcium, high in sugar-sweetened beverages, high in processed meat, low in milk and high in red meat” [13].

The nutrition transition documented in many LMICs is closely associated with the development of many NCDs [14,15]. This transition is described as a major change in diet from a traditional pattern which is largely composed of unrefined grains, legumes, fruit, and vegetables towards one of refined starches, added sugars, and animal products, fats and oils combined with a reduced intake of fruit, vegetables, legumes, nuts, and seeds [14,15], which has also been described as an obesogenic dietary pattern [4]. The obesogenic dietary pattern has been associated with several potential drivers, including economic growth, fast urbanization, and increase in the production and consumption of highly processed foods, as well as socio-economic and lifestyle characteristics [16]. Numerous studies have shown that these ultra-processed foods do not have the same nutritional benefits as unprocessed foods [17].

The analysis of dietary patterns for investigation of diet–disease interactions was introduced in the 1980s [18,19,20,21]. Dietary patterns provide a broader picture of food and nutrient consumption that reflects not only the effect of individual nutrients, but also the contribution of dietary variety and interactions between dietary components [22]. According to Malekua et al. [23], identifying dietary patterns that consider the overall eating habits, rather than focusing on individual foods or simple counts of consumed foods, better helps to understand the combined effects of dietary components. This information can be translated into suitable dietary guidelines for children to prevent the establishment of obesogenic dietary patterns, while promoting healthy dietary patterns [24].

Healthy dietary patterns have been described as a “diet [that] largely consists of vegetables, fruits, whole grains, legumes, nuts, and unsaturated oils, includes a low to moderate amount of seafood and poultry, and includes no or a low quantity of red meat, processed meat, added sugar, refined grains, and starchy vegetables”; thus, diverse and nutrient-dense [25]. As a result, many countries have adopted food-based dietary guidelines [26] to assist children and adults in making healthy food choices, including South Africa [27,28].

The aim of the present study was to characterise the dietary patterns of children aged 1–<10 years who were studied during the Provincial Dietary Intake Survey (PDIS) in 2018, and to investigate socio-demographic predictors of the identified dietary patterns, as well as associations between dietary patterns and stunting and overweight/obesity. In South Africa, little data are available regarding the diet of children and how the nutrition transition has affected their dietary intake. The only national survey in children was undertaken in 1999, the National Food Consumption Survey (NFCS), with no follow up for comparison to show trends and changes in diet [8]. The PDIS study was a follow-up of the NFCS study in two rapidly urbanizing and economically active provinces, Gauteng and the Western Cape, to investigate dietary intake and growth status in children aged 1–<10-years [8].

## 2. Materials and Methods

### 2.1. Study Area

The two provinces selected were Gauteng (GTG) and the Western Cape (WC), because they are the most rapidly urbanizing and wealthiest provinces with extensive migration from rural areas to cities in search of jobs and a better quality of life [29].

### 2.2. Structure of the Sample and the Sampling Procedure

The detail of sample structure and sampling procedure has been described elsewhere [7]. Briefly, six strata were identified, namely two provinces (GTG and WC), with each having three areas of residence: urban formal, urban informal, and rural areas. Enumerator areas (EAs) were identified in each stratum. A stratified two-stage sample design was used with a probability proportional to the size sampling of EAs at the first stage, and systematic sampling of households within the EAs at the second stage. The formula included below was used to determine the number of households per stratum, for six domains:

N = Deft^2^ × {[(1/P) − 1]/a^2^}/(R_1_ × R_2_ × d)

where

N = the number of households per sampling stratum, taking non-response into account was calculated to be N (=175);
Deft (=1.3) is the design effect;
P (=0.21) is the estimated proportion of children classified as stunted;
a (=0.2) is the desired relative standard error;
R1 (=0.96) is the individual response rate;
R2 (=0.89) is the household gross response rate;
d (=1.06) is the number of eligible individuals per households [7].

The number of eligible individuals per households (d = 1.06) was calculated as the average number of children aged 1–<10 years per household. It was proposed to survey 175 × 6 strata, or 1050 households. Precision of estimates across regions (rural, urban informal, and urban formal) was ensured by including a minimum of 50 interviews per stratum [7]. Since the sample sizes of GTG rural, WC rural, and urban informal were less than 150, we increased sampling accordingly to ensure sufficient observations per cell in each age group, with the proposed sample size then being 1050 + 218 = 1268. A total of 84 EAs were selected from the six strata, 25 formal residential, 10 informal residential, and 11 rural EAs in GTG, as well as 18 formal residential, 10 informal residential, and 10 rural EAs in the WC.

### 2.3. Selection of Households and Children within Households

Maps of primary sampling units were generated and passed on to fieldwork teams. The total number of households (HHs) in each EA and a listing of eligible HHs was compiled for each EA, which served as the sampling frame for the selection of HHs. A maximum of 16 HHs were selected per EA based on a predetermined fixed interval (calculated to be specific to each EA) starting from a randomly determined point. A backup sampling frame was constructed in each EA by asking members of the 16 selected HHs to identify nearby HHs with women and children of the appropriate age of 1–<10 years old.

One child in each randomly selected HH was included in the survey. If more than one child in the prescribed age interval was present in the HH, then all eligible children in the HH were numbered in age order for random selection of one child using a “Random Number Table” designed for this purpose.

Inclusion criteria were as follows: children aged 1–<10 years (12–119 months) old; male or female; availability of a parent/primary caregiver to provide consent; and availability of a parent/primary caregiver to assist with completion of the research questionnaires. Children who were mentally or physically handicapped; who were on a prescribed diet for a childhood disease, e.g., Type 1 diabetes, phenylketonuria, and other conditions; who were ill at the time of the visit or were ill during the past 24 h; whose mothers/caregivers were unable to respond, or appeared to be incapable of responding or providing reliable information; whose mother/caregiver was under the influence of alcohol/drugs or was under 15 years old were not eligible for participation.

Sampling weights were calculated to adjust for the oversampling in the rural and urban informal areas and the number of children in the 1–<3, 3–<6, and 6–<10 year age groups, bearing in mind the survey design. The final post hoc stratification weighting reflects the census population of the Western Cape and Gauteng provinces [30]. The three age groups were demarcated to reflect children who are in the first 1000 days of life (children in their third year, but not yet three years old, were included in this group), older preschool children, and primary school-aged children.

### 2.4. Fieldwork Teams

Fieldwork in each province was led and managed by a registered dietitian (fieldwork coordinators). Research teams in the two provinces included a team leader and two pairs of experienced field workers. Team leaders and field workers received a week-long extensive training session using a manual developed for the purpose of the study. Training was facilitated by researchers experienced in administration of sociodemographic and dietary questionnaires, as well as the fieldwork coordinators. Training of the WC fieldwork teams took place in Cape Town and was attended by the GTG fieldwork coordinator. Subsequently, training of GTG fieldworkers took place in Johannesburg, which was co-facilitated by the WC fieldwork coordinator to contribute to data fidelity. After each training module, the field workers practiced using the questionnaires through role play sessions with each other. At the end of the week, the field workers completed a practical and written test based on case studies. Field workers who did not achieve a certain percentage were not selected.

### 2.5. Measures

#### 2.5.1. Socio-Demographic Questionnaire

The socio-demographic questionnaire included questions (predictors) which could impact the dietary intake and health outcomes of children and were based on the child, family, household, and environment. Questions about the child included birth date, gender, primary caregiver, and whether they attended a creche or preschool facility. Questions about the family and household were head of household, marital status of mother, education and employment status of mother and father, type of house, availability of electricity or other energy devices, source of drinking water, type of toilet, and household density. These variables were selected as they were used in the National Food Consumption Survey (1999), and many were found to be significant predictors of nutritional status at the time [31].

A wealth index was calculated as indicated by the World Bank [32] and applied in the 2016 South Africa Demographic and Health Survey [33]. Iterated principal factor analysis was used to estimate relative wealth, and this estimation is based on items loading on the first factor. The first factor contributes to a wealth index that assigns a larger weight to assets that vary the most across households, so that an asset found in all households is given a weight of zero. The wealth index in this study was based on amenities available in the home and environment [7].

Hunger (food security) was measured using the Community Childhood Hunger Identification Project (CCHIP) questionnaire [34]. This questionnaire measures household, child, and individual-level food security. The scale comprises eight questions and a score of one is given for affirmative answers. A total score of 5–8 indicates the presence of food shortage (hunger) in the household, a score of 1–4 reflects risk of hunger, and a score of zero indicates that the house is food-secure (no hunger).

#### 2.5.2. Dietary Intake

A 24 h recall was conducted with each participant to determine dietary intake using the multiple pass method [35] (details on the application of this method have been described elsewhere, Steyn et al. [36]). The literature indicates that the accuracy of reporting one’s own dietary intake in younger children is not good, but that it improves between the ages of 8 and 12 years [37]. Consequently, in this study, all dietary interviews took place in the presence and with the input of the mother/primary caregiver. For 1–6-year-old children, the mother/caregiver reported on the intake of the child on the previous day with no input from the child. For 7–<10-year-old children, the mother/caregiver and child were interviewed together to record the dietary intake during the prior 24 h. If the child had been at a day care centre the previous day, the centre was visited by the fieldworker and the meals and portion sizes determined for the 24 h in question. All weekdays and Sundays were covered proportionally by each team to ensure that potential variation due to day of the week was captured.

**Figure 1:**
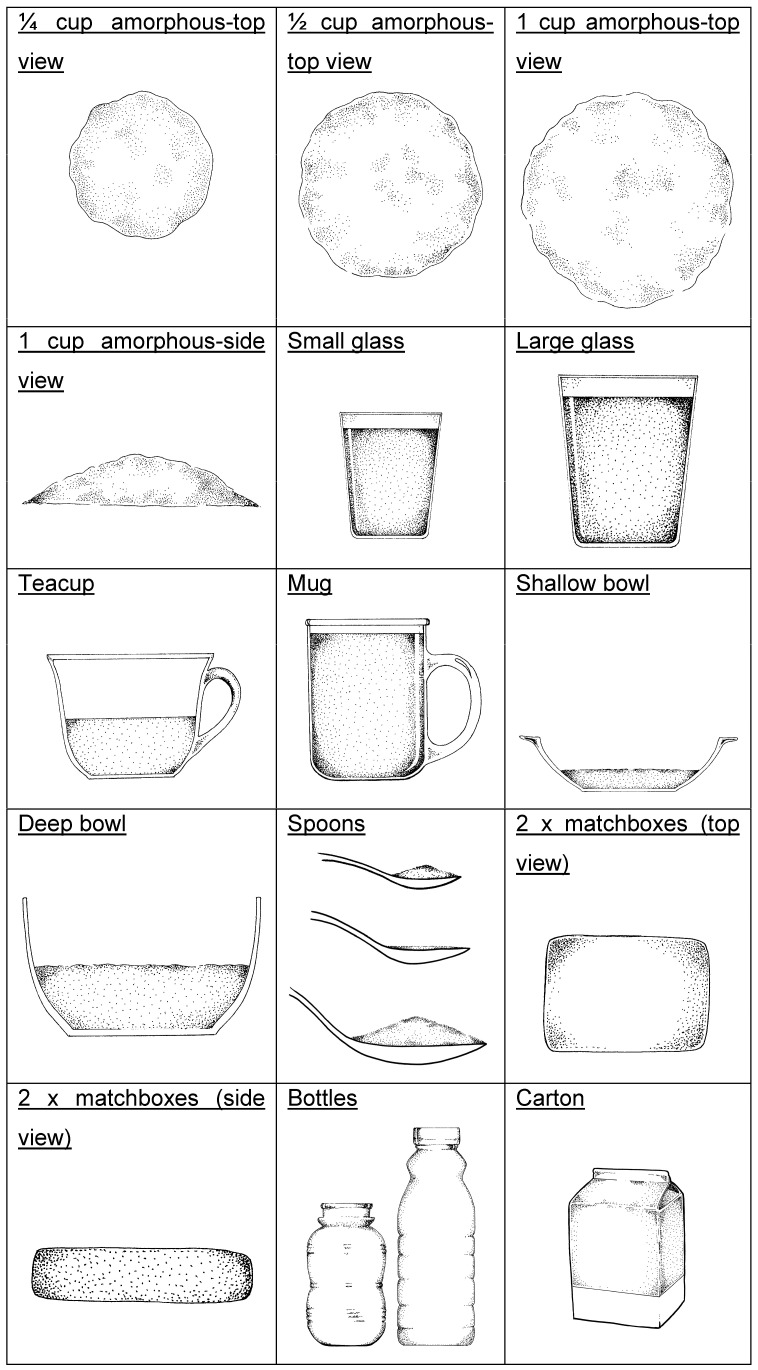
Life-size generic models used in the DAEK [38].

Portion sizes were obtained using a booklet adapted from the Dietary Assessment and Education Kit (DAEK) [38]. The booklet comprises life-size sketches of generic household utensils and crockery (Figure 1) and life-size portions of actual foods, e.g., different slices of bread varying in size and thickness, to make estimations of portion size as accurately as possible. The sketches were validated in adolescents [39]. Generic three-dimensional food models made from flour were also used to assist in recording volume measures such as porridge and rice.

Breast milk consumption was quantified by asking mothers whether their child was still receiving breast milk and, if yes, the number of feeds the child received during the previous 24 h. Based on the study by Neville et al. [40], we used an estimate of 100 mL per feed to calculate the volume of breast milk consumed per day.

A common concern with a single 24 h recall is the day-to-day variation in the diet of free-living populations. The National Cancer Institute (NCI) method [41,42] that was developed to distinguish within-person from between-person variation accounts for extreme intakes, including zero intake, allows for adjustment for covariates and association analyses, and was applied in this study to estimate the usual dietary intake from repeated 24 h dietary recall assessments on a subsample of 148 (second recall) and 146 (third recall) children (details on the application of this method in this research have been described elsewhere) [36].

After completion of an EA, the questionnaires were verified by the two registered dietitians who managed the fieldwork in the two provinces for quality control purposes. The 24 h recalls were coded by these dietitians using the South African Food Composition Tables (SAFCTs) [43], each coding the recalls collected in their allocated province. Where necessary, codes were confirmed in consultation with the rest of the research team to ensure uniform decisions and coding of foods.

Food items were allocated to 30 food parameters based on the similarity of nutrient profiles in the allocated SAFCT food codes [43] (Table 1).

### 2.6. Data Management and Analysis

All data were captured centrally by two experienced researchers. Data analyses were conducted using SAS Version 9.4, SAS for Windows (SAS Institute, Carry, NC, USA). Frequencies were tallied for the socio-demographic variables which were compared between the WC and GTG using the Rao–Scott chi-squared test, incorporating the complex survey design.

Dietary pattern analysis was conducted within each of the designated age groups, namely 1–<3-year-olds, 3–<6-year-olds, and 6–<10-year-olds using principal factor analysis with varimax rotation [44]. It is evident from other research that the dietary units of analysis used for dietary pattern identification include percentage of total daily energy contribution (kJ) of each food group/item [45], or daily amount consumed from each food group/item in grams [46], or daily frequency of consumption of a food group/item [47]. We conducted principal factor analyses using each of these options. After consideration of the Kaiser–Meyer–Olkin (KMO) statistic, which tests the appropriateness of applying principal factor analysis to the dataset (post hoc sampling adequacy) [48], as well as the percentage total variance explained by the identified patterns, it was evident that the frequency of consumption of food groups/items was the most appropriate dietary unit to use for our dataset.

As the frequencies of intake of the 30 food parameters calculated for this research (Table 1) were not normally distributed, the data were normalised using Blom’s transformation [49]. Ricci et al. explain that this transformation is particularly suitable to normalise and standardise food or nutrient intakes before principal factor analysis is conducted [50]. The decision on the number of dietary patterns to be retained was based on the visual inspection of the scree plot, eigenvalues of >1.5, and interpretability of the pattern, as was conducted by Faber et al. [51]. No golden rule on the cut-off for the exclusion of food parameters in the principal factor analysis, using the pattern (factor) loadings, has yet been set. We retained food parameters with a loading of >0.3 and <−0.3 for interpretation and naming of each dietary pattern, which is in line with the cut-offs used by several other researchers [50,51,52]. Factor scores were generated for each food parameter using the loading of all 30 food parameters on each factor pattern. Higher factor scores reflect greater adherence of the food parameter to the specific pattern.

Predictors of dietary patterns were identified by constructing multiple regression models with backward elimination. As five patterns were retained in each age group, a total of 15 regression models were constructed. The dietary pattern scores were standardized-dependent variables with zero means and standard deviations of 1 unit. The socio-demographic variables outlined above, as well as province (the WC and GTG) were the independent variables in these analyses. The parameter estimate, standard error (SE), as well as the *p*-value for the independent variables that showed a significant association with the outcome variables that were retained in the final models are reported in the results. Variance inflation factors (VIFs) were calculated and were all less than 1.6, indicating the absence of multicollinearity.

The association between dietary patterns and anthropometric variables was investigated using logistic regression with (1) BAZ > 2SD and (2) HAZ < −2SD as dependent variables, with the pattern score as independent variable while controlling for age, gender, and province.

## 3. Results

### 3.1. Results for Sociodemographic Profile of HHs

Data on the socio-demographic profile of HHs included in the study are shown in Table 2. The sample comprised 49.3% of boys and 50.7% of girls. For 70.4% of the children, the primary caregiver was the mother, while the head of the household was mostly the father (39.7%) or the grandmother (24.0%). Fifty-three percent of mothers did not complete grade 12 compared with 29.1% of fathers. Significantly more mothers were employed in the WC compared with GTG (38.4% vs. 22.4%), while 65% of all fathers were employed. In the WC, 68.0% of the sample were of mixed ancestry, while in GTG 97.8% were black African. The majority of the sample were urban formal residents (88.2%). Food insecurity was present in 20.7% of the households.

### 3.2. Results for 1–<3-Year-Old Children

The five dietary patterns and significant socio-demographic predictors for 1–<3-year-olds are shown in Table 3. The total variance explained by the five patterns in this age group is 31.7%.

Maize porridge had a very high (PL = 0.84) positive pattern loading (PL) on the first dietary pattern. Soup/sauces also loaded positively (PL = 0.44), while dairy and refined carbohydrates loaded negatively. This pattern was labelled the “***Pap & sauce pattern***” Pap is the term commonly used for maize porridge which can be eaten soft, stiff, or crumbly as a starch. Predictors for greater adherence to this pattern were living in Gauteng and being at risk of or experiencing hunger. Predictors of lesser adherence were having an aunt or uncle as head of the household, a mother with a grade 12 qualification, a father who has a post grade 12 qualification, and a greater wealth index.

The two food parameters that had the highest positive loading on the second pattern were tea/coffee (PL = 0.74) and sugar (PL = 0.72) and this was labelled the ***Tea/coffee and sugar pattern***. Fats and oils high in saturated fat and legumes also loaded positively on this pattern (PL < 0.6). Predictors of greater adherence to this pattern were a higher wealth index and being at risk of hunger. Being looked after by a sibling or aunt predicted lesser adherence.

Foods that comprised a combination of refined carbohydrates and fat, e.g., crisps or savoury biscuits (PL = 0.52) and foods that comprise a combination of refined carbohydrates, sugar, and fat, e.g., cake, ice cream, and chocolate (PL = 0.5), brown bread (PL = 0.42), SSBs (PL = 0.41), and fruit (PL = 0.36) loaded positively on this pattern, and was labelled the “***Mostly unhealthy snack pattern***”. Baby foods loaded negatively on this pattern. Predictors of greater adherence to this pattern were having a mother with a grade 12 qualification and a father with a post grade 12 qualification. Having a grandmother as primary caregiver predicted lesser adherence.

White bread had the highest positive loading on the fourth pattern (PL = 0.65), with processed meat (PL = 0.53), miscellaneous items, e.g., Marmite, Bovril, fish paste, and condiments (PL = 0.36), as well as eggs (PL = 0.32) also loading positively on this pattern, and was labelled the “***White bread & topping pattern***”. Significant predictors of lesser adherence to this pattern were living in Gauteng, being looked after by a sibling or aunt, and having an obese mother. There were no predictors of greater adherence.

Unsaturated fats and oils had the highest positive loading (PL = 0.6) on the fifth pattern. Vegetables (all except starchy vegetables) (PL = 0.41) and fish (PL = 0.31) also loaded positively, while poultry (PL = −0.35) and sweets (PL= −0.55) loaded negatively on this pattern, and were labelled the “***Healthy pattern***”. Predictors of greater adherence to this pattern were living in Gauteng, having an overweight mother or obese mother, and a higher wealth index. Having a grandparent as head of the household predicted lesser adherence.

Starchy vegetables combined with fat, e.g., “slap chips” (French fries) did not load on any dietary pattern in this age group.

There were no significant associations between dietary patterns and HAZ or BAZ variables (results not shown in a table).

### 3.3. Results for 3–<6-Year-Old Children

Dietary patterns and significant socio-demographic predictors for 3–<5-year-olds are shown in Table 4. The total variance explained by the five patterns in this age group is 30.5%.

Tea/coffee (PL = 0.85) and sugar (PL = 0.82) had very high positive loadings on the first dietary pattern. Fats and oils (saturated PL = 0.49 and unsaturated PL = 0.31 fats/oils), as well as brown bread (PL = 0.33) also loaded positively on this pattern, which was labelled the “***Tea/coffee, sugar & sandwich pattern***”. There were no significant socio-demographic predictors of this pattern.

The second pattern was labelled the “***Unhealthy pattern***” as all food parameters that loaded positively on it were deemed to be unhealthy. These items are white bread (PL = 0.65); starchy vegetables combined with fat, e.g., “slap chips” (French fries) (PL = 0.55); foods which combine refined carbohydrates with animal protein and fat, e.g., pies, “vetkoek”, pasta dishes and pizza (PL = 0.41); foods which combine refined carbohydrate with sugar and fat, e.g., cake, doughnuts, ice cream, and chocolates (PL = 0.41); and processed meat (PL = 0.36). Predictors of greater adherence to this pattern were having a grandparent as head of the household, a higher wealth index, and having a mother who is employed. Living in Gauteng was a predictor of lesser adherence.

The food parameter, RC-other, which includes rice and pasta, had the highest loading (PL = 0.71) on the third pattern. Starchy vegetables (PL = 0.48) and poultry (PL = 0.43) also loaded positively on this pattern, while maize porridge loaded negatively (−0.53). It was labelled the “***Starch & poultry pattern***”. Being looked after by a sibling or aunt, living in Gauteng, and having an overweight mother were predictors of lesser adherence to this pattern. There was no significant predictor of greater adherence.

Four food items that could typically be consumed as part of breakfast loaded positively on the fourth pattern, including dairy (PL = 0.62), fruit (PL = 0.57), cheese (PL = 0.46), and refined breakfast cereal (PL = 0.46). This pattern was labelled the “***Breakfast pattern***”. Predictors of greater adherence to this pattern were being a girl, a higher wealth index, and having a mother with a post grade 12 qualification, a father with a grade 12 or a post grade 12 qualification, an employed mother, and an obese mother. Predictors of lesser adherence were living in Gauteng, being looked after by a grandmother, and having an employed father.

Legumes (PL = 0.41), vegetables (all except starchy vegetables) (PL = 0.41), and miscellaneous items, e.g., Bovril, marmite, fish paste (PL = 0.40) loaded positively on the fifth pattern, while unrefined carbohydrates loaded negatively (PL= −0.64). This pattern was labelled the “***Vegetable & legume pattern***”. Predictors of greater adherence to this pattern were living in Gauteng and the presence of hunger in the household. There was no significant predictor of lesser adherence.

Soups/sauces, as well as foods that combine refined carbohydrates and fat, e.g., crisps (any type) and salty biscuits did not load on any pattern in this age group.

There were no significant associations between dietary patterns and HAZ or BAZ variables (results not shown in a table).

### 3.4. Results for 6–<10-Year-Old Children

Dietary patterns and significant socio-demographic predictors for 6–<10-year-olds are shown in Table 5. Total variance explained by the five patterns in this age group is 31.37%.

Foods that combined refined carbohydrates with fat, e.g., crisps and salty biscuits, had the highest loading on the first pattern (PL = 0.47), followed by SSBs (PL = 0.44), fruit (PL = 0.41), unrefined cereals (PL = 0.4), and sweets (PL = 0.36), while fish (PL = −0.33), legumes (PL = −0.39), and maize porridge (PL = −0.5) loaded negatively. As only two healthy food items loaded positively (versus three unhealthy items) and three healthy items loaded negatively on this pattern, it was deemed to be more reflective of unhealthy eating. It was labelled the “***Mostly unhealthy pattern 1***”. Predictors of greater adherence to this pattern were having a father with a grade 12 qualification, a higher wealth index, and having an obese mother. Predictors of lesser adherence to this pattern were living in Gauteng, being at risk of experiencing hunger, and having hunger present in the household.

Tea and/or coffee (PL = 0.85), sugar (PL = 0.82) and dairy loaded positively on the second pattern and this was labelled the “***Tea/coffee, sugar and dairy pattern***”. Predictors of lesser adherence to this pattern were the mother being the head of the household, being looked after by a sibling or aunt, being a girl, and living in Gauteng. There was no significant predictor of greater adherence to this pattern.

Food parameters that loaded positively on the third pattern were mostly unhealthy, including refined breakfast cereals (PL = 0.53); foods which combined refined carbohydrates with animal protein and fat, e.g., pies, “vetkoek”, pasta dishes, and pizza (PL = 0.34); and foods which combined refined carbohydrates with sugar and fat, e.g., cake, doughnuts, ice cream, and chocolates (PL = 0.32), with red meat being the exception (PL = 0.44). As two healthy food parameters, non-starchy vegetables and poultry also loaded negatively on this pattern, which was labelled the “***Mostly unhealthy pattern 2***”. Predictors of greater adherence to this pattern were a higher wealth index and having a mother with a post grade 12 qualification. Predictors of lesser adherence were living in Gauteng and having hunger present in the household.

White bread had a high loading PL = (0.84) on the fourth pattern together with unsaturated fats and oils (PL = 0.48) and processed meat (PL = 0.42), while brown bread loaded negatively (PL = −0.51). This pattern was labelled the “***White bread & processed meat pattern***”. The only significant predictor for this pattern was living in Gauteng, which predicted lesser adherence.

The fifth pattern was labelled the “***Non-maize pap or bread starch pattern,***” as “other” refined carbohydrates, e.g., rice and pasta (PL = 0.68) and starchy vegetables (PL = 0.46) loaded positively on this pattern. Predictors of greater adherence to this pattern were having an aunt or uncle as head of the household, being looked after by a grandmother, being at risk of experiencing hunger, and having hunger in the household. Predictors of lesser adherence were having an employed father and living in Gauteng.

Soups/sauces, and starchy vegetables that were combined with fat, e.g., “slap chips” (French fries), did not load on any pattern in this age group.

There were no significant associations between dietary patterns and HAZ or BAZ variables (results not shown in a table).

## 4. Discussion

A review of the literature shows that there are not many studies that report on the dietary patterns in children. In the present study, we set out to examine dietary patterns and socio-demographic predictors thereof in 1–<10-year-old children in two economically active provinces in South Africa. We also investigated associations between identified patterns and anthropometric indicators in the children. The results show that the dietary patterns were far from ideal, with no associations with anthropometric indicators. Predictors of both healthy and unhealthy patterns related to having a higher socio-economic status and an obese mother.

Tea (mostly rooibos tea) with sugar, but not so much coffee, is a drink that has previously been found to load strongly on dietary patterns in children under 2 years of age in lower income areas in in KwaZulu Natal and the North West province in South Africa (using a single unadjusted 24 h recall for dietary pattern analysis) [51]. Moreover, these researchers found that, in their 18–24-month-old group, sugar also had a high loading on the tea pattern. This indicates that tea was taken with sugar and the researchers speculated that mothers were substituting breast milk/formula milk with tea as children grew older [51], despite the recommendation in the paediatric food-based dietary guidelines that tea, coffee, and sugary drinks should be avoided [27]. Our results confirm that a *tea/coffee–sugar* pattern seems to be common in young children in South Africa as a *Tea/coffee and sugar* pattern emerged as one of the two strongest patterns in each age group, with pattern loadings (PLs) for tea/coffee and sugar being >0.8 in the two older groups and >0.7 in the 1–<3-year-old group.

Dairy also loaded on the *Tea/coffee and sugar* pattern in the 6–<10-year-olds, but not in the two younger age groups. This may indicate that tea is not necessarily given with milk in the younger age groups, reducing the potential nutrient density of the pattern in terms of quality protein, calcium, and other micronutrients. The practice of feeding children sugar in combination with black tea was also reported for children in Kenya and Tanzania [52]. However, in the 4–<6-year-olds, brown bread and fats (saturated and unsaturated), loaded on the tea/coffee and sugar pattern, indicating that the drink may be accompanied by a sandwich made form healthy bread with butter/margarine as a spread, increasing nutrient density. Predictors of the *Tea and coffee pattern* were contradictory, with indicators of both a higher socio-economic and a hunger profile being contributors to greater adherence to the *Tea/coffee and sugar* pattern.

Patterns that reflect the energy-dense, nutrient-poor Western dietary pattern [14] were prominent in the study sample. Pattern 3 (*Mostly unhealthy snacks*) in 1–<3-year-olds comprised unhealthy snacks such as crisps, salty biscuits, cake, sweet biscuits, ice cream, chocolate, and SSBs (fruit also loaded on this pattern, but with a lower PL). Pattern 4 (*White bread and toppings*) in this age group comprised a sandwich on white bread with mostly unhealthy toppings (processed meat, salty spreads). Pattern 2 (*Unhealthy foods and snacks*) in the 4–<6-year-old age group was deemed to be unhealthy and comprised white bread, “slap chips,” items such as pies, “vetkoek”, pasta dishes, pizza, cake, as well as the unhealthy snacks mentioned for the 1–<3-year-olds. The strongest pattern (*Mostly unhealthy 1*) in the 6–<10-year-old group comprised unhealthy snacks such as crisps, salty biscuits, SSBs, and sweets (fruit and unrefined breakfast cereal also loaded on this pattern, but with lower PLs). Pattern 3 (*Mostly unhealthy 2*) in this age group comprised unhealthy meal items such as refined breakfast cereals and foods/items such as pies, “vetkoek”, pasta dishes, and pizza, as well as the mentioned unhealthy snacks. Pattern 4 (*White bread and processed meat*) in this age group comprised white bread, a saturated/unsaturated fat (margarine) spread, and processed meat.

Unhealth dietary patterns consumed by South African children have been reported by a few groups. Hooper et al. [53] indicated that the diets of 8–13-year-olds in urban areas in the Western Cape included unhealthy items such as fried potatoes, sausages, tinned fruit salad, custard, and jelly. Faber et al. [51] described a *More westernized pattern* in children under 2 years of age in KwaZulu Natal and the North West province, which was found to be positively associated with unhealthy nutrients such as cholesterol and saturated fat, emphasizing the importance of interventions to address unhealthy food choices. White bread flour in South Africa is fortified with eight micronutrients [54], but the fortification mix does not include calcium; vitamins C, D, and E; and other biologically active compounds found in unrefined cereals, fruit, and vegetables. White bread with a margarine spread and polony seems to be a recurring pattern in young children in the Western Cape, either served as a meal at home or included in the school lunch box [55]. Together with refined breakfast cereals, white bread may reflect the presence of poor food choice patterns, as they were combined with unhealthy snacks and foods in the patterns we identified.

A higher socio-economic status (higher wealth index, father and/or mother with grade 12/post-grade 12 qualification, and mother employed) were significant promoters of the unhealthy patterns identified in the present study. Temple and Steyn [56] and Heady et al. [57] showed that healthy foods such as milk, animal proteins, vegetables, and fruits, are more expensive than unhealthy foods. However, in their comparison of relative caloric prices (RCPs) of healthy and unhealthy across income levels and continents, Heady et al. [57] also categorized soft drinks, fruit juice, and salty snacks as expensive, and processed meats as very expensive. The Health Promotion Levy on sugary beverages was legislated 2017, with the aim of reducing SSBs consumption [58]. If households were to continue purchasing these items, as is evident from the PDIS results where these drinks were the fifth most consumed item (31% in 1–<3-year-olds; 42% in 3–<6-year-olds; 50% in 6–<10-year-olds) [36], it would come with the extra cost. The continued use of SSBs in South African communities is further illustrated in the results of the household inventory conducted by O-Halloran et al. [55] in low-income households in the Cape Town Metropole, where fizzy drinks were present in 66.6% of surveyed households. Of note is that fruit, which is classified as expensive [56,57], also loaded on Pattern 3 in the 1–<3-year-olds and Pattern 1 in the 6–<10-year-olds, albeit with lower PLs than the unhealthy items. Socio-demographic predictors of a *Sweet tooth dietary pattern* in Ghanaian adolescents also included household wealth, living with parents, and going to school with pocket money [59]. Money taken to school is usually spent at school tuckshops, where mostly unhealthy items, including crisps, sweets, chocolates, and fizzy SSB are sold to children in higher and lower socio-economic areas [55,60].

Each of the three age groups had a dietary pattern that was composed mainly of one or a combination of starches. This could reflect the South African Food-Based Dietary Guideline of making starchy foods part of most meals [61]. Maize porridge is typically given to young children in South Africa, especially in under 2-year-olds, either as a soft porridge at breakfast and/or in a stiffer consistency at lunch and/or supper with/without a sauce/soup containing some form of meat and/or vegetables [36,51]. It was thus not surprising that the strongest pattern in the 1–<3-year-olds in the youngest age group in the current study comprised maize porridge as the key starch and a soup/sauce (*Maize pap and soup/sauce pattern)*. The nutrient density of this pattern may be acceptable if combined with a sauce/soup containing a quality protein, because maize meal is also fortified with eight micronutrients [54]. However, it is a concern that dairy does not load on this pattern in this age group. Pattern 3 (*Starches and poultry*) in the 4–<6-year-olds and Pattern 5 (*Starches*) in the 6–<10-year-olds comprised rice, pasta, and starchy vegetables, with poultry also loading on Pattern 3 in the 3–<6-year-old group. Starch-based diets could be low in nutrient density if they do not include a fortified cereal and are not combined with quality protein, fruit, and vegetables. Being at risk of or experiencing hunger promoted the starchy patterns in the youngest and oldest age group, while there were no promotors of the pattern in the 4–<6-year-old group. Starchy dietary patterns have been shown to be linked to poverty [62,63].

Patterns that included mostly healthy food items were Pattern 5 (*Vegetables and Fish*) in the 1–<3-year-old group and Patterns 4 (*Breakfast items*) and 5 (*Legumes and Vegetables*) in the 4–<6-year-old group. Pattern 5 in the youngest age group included unsaturated fats/oils, vegetables (except starchy vegetables), and fish, with the fats/oils most probably used in the preparation of the fish and vegetables. Healthy food items that loaded on Pattern 4 (*Breakfast items*) in the 3–<6-year-old group were dairy, fruit, and cheese. However, refined breakfast cereal, which is typically not classified as healthy, also loaded on this pattern. Based on the profiles of expensive versus cheap foods outlined by Temple et al. and Heady et al. [56,57], these two patterns can be categorized as expensive. The promotors of greater adherence to these patterns were higher WI (both patterns), higher qualified mother (both patterns), and father and an employed mother, confirming that income plays a role in the establishment and maintenance of these mostly healthy patterns. Pattern 5 in the 4–<6-year-olds included legumes and vegetables (excluding starchy vegetables), which are classified as healthy food choices [64,65]. However, salty spreads and condiments also loaded equally on this pattern. Faber et al. [51] identified a *Rice and legume pattern* in in 12–17-month-olds from low socio-economic populations in South Africa in Kwa-Zulu Natal and the Northwest province, which was positively associated with fibre, plant protein, and polyunsaturated fat [51]. Of note is that, for some children a pattern consisting of either legumes and vegetables, or rice and legumes, this may not be combined with high-quality protein which would not sustain optimal growth and development. Confirming this notion, Pisa et al. [66] found a positive association between BMI-for-age z-scores and a dietary pattern characterized by animal products and a second pattern comprising starch and folate. Our results show that hunger in the household promoted greater adherence to the *Legume and vegetable pattern* in this age group, suggesting that there is a possibility that quality proteins may be lacking.

It is important to note that not a single pattern that included mostly healthy items emerged in the 6–<10-year-old group. This illustrates a potential decrease in dietary quality with increasing age among primary school children. The results on adequacy of micronutrient intake in the PDIS sample confirms that 6–<10-year-olds had a lower intake of calcium, phosphorus, zinc, and vitamin C than the younger age groups [67]. Dietary pattern identification using data from a quantified food frequency questionnaire (recall period past 7 days) and principal component (PC) analysis in 9–11-year-old children in urban areas from 12 countries across the world (*n* = 7199) resulted in two strong components, namely a *Healthy pattern* and an *Unhealthy pattern*. The *Healthy pattern* included dark green vegetables, orange vegetables, other vegetables, berries, and fruits while the *Unhealthy pattern* included fast foods, fried food, French fries (“slap chips”), and SSBs. The researchers concluded that the same “healthier” and “unhealthier” foods tend to be consumed in similar combinations among 9–11-year-old children in different countries, despite variation in food culture, geographical location, ethnic background, and economic development [68].

A few studies in children and adolescents have shown associations between dietary patterns and undernutrition outcomes. Three patterns that included a quality animal protein were identified in children aged < 5 years living in rural Burkina Faso, but the *Leaves-based diet* did not result in improvements in wasting and stunting (dietary method used: semi-quantitative food frequency questionnaire; recall period—past 7 days) [69]. Analysis of dietary data for children younger than 5 years of age from the Demographic Surveillance System conducted in Kwale County, Kenya, showed that the *Traditional pattern* (minimal animal protein) showed a higher risk for stunting compared with the *Protein-rich pattern* (dietary method used: semi-quantitative food frequency questionnaire; recall period—past month) [70]. A greater adherence to a *Dairy, vegetable, and fruit pattern* was found to be associated with increased HAZ and reduced risk of stunting in younger than 5-year-olds in Ethiopia. However, no significant associations between the *Egg, meat, poultry, and legume pattern* with HAZ and stunting were found (dietary method: single 24 h recall of frequency of intake of seven or nine food groups depending on age) [23]. In 6–19-year-old Nigerian children and adolescents, a *Traditional dietary pattern* (containing mainly cereals/starchy food and legumes, and thus no quality animal protein) increased, while a diversified dietary pattern (containing all food groups) reduced the odds for thinness (dietary method: food frequency questionnaire; recall period—past month) [70]. In the present study, at least one of the five dietary patterns identified for each of the three age groups included a quality animal protein. Egg loaded positively on pattern 4, and fish on pattern 5 in 1–<3-year-olds; poultry on pattern 3, and dairy and cheese on pattern 4 in 3–<6-year-olds; and dairy on pattern 1, and red meat on pattern 2 in 6–<10-year-olds. Based on findings by others, the expectation was that one or more of these patterns would protect against stunting. However, we did not find any associations between stunting and any of the dietary patterns in the three age groups.

Associations between energy-dense dietary patterns and overweight/obesity have also been reported, albeit mostly in adolescents. Keding et al. [52] stated that an average of 10% of urban children in Kenya and Tanzania were overweight or obese. According to these researchers, this is mainly due to a *Purchase dietary pattern*, which is dominated by bought and processed foods. In the Nigerian study mentioned above, the *Traditional dietary pattern* did not only promote thinness, but also increased the odds of being overweight or obese, reflecting a double burden of malnutrition linked to this dietary pattern [71]. As for stunting, no associations between any of the dietary patterns and overweight/obesity were found in the current study.

Finally, in the present study, the BMI of the mothers showed interesting associations with the dietary patterns of their children. In the 1–<3-year-olds, an obese mother predicted greater adherence to the *Healthy pattern* (unsaturated fats/oils, vegetables, and fish) and lesser adherence to the *White bread and topping pattern* (processed meat and salty spreads), and in the 3–<6-year-olds greater adherence to the mostly healthy *Breakfast item pattern*. In the 6–<10-year-olds, an obese mother predicted increased adherence to the strongest pattern, which was *Mostly unhealthy pattern 1.* Various elements of the energy-dense, low-nutrient Western dietary pattern have been linked to obesity in adults [72,73,74]. From their systematic review of risk factors for overweight and obesity within the home environment of preschool children in sub-Saharan Africa, Kwansa et al. concluded that the home food environment, through the types of foods offered, and greater maternal BMI, were key aspects contributing to overweight and obesity among pre-schoolers [74]. Our results may indicate that these effects were not yet in play in the 1–<6-year-olds in the Western Cape but affected the dietary patterns of the 6–<10-year-olds.

### Limitations

Although the dietary data in the present study were collected using an adjusted 24 h recall to remove intra-individual variability, the recall period does not necessarily reflect the dietary patterns of children in early life. This may explain the lack of association between dietary patterns and anthropometric outcomes in the present study. We suggest that an investigation of the dietary intake of a cohort of children from one to 18 years of age may provide better insights into long-term dietary patterns and associations with anthropometric indicators and other health outcomes. However, the feasibility of this type of study design in an LMIC is questionable due to limited funding and resources, as well as the logistics of tracing often migrating children over such a period of time. Periodic cross-sectional surveys add value in terms of insights into the dietary patterns of children that can be used in intervention planning and assessment. Despite the limitation linked to the recall period, the dietary patterns that emerged are in line with expectations when considering the nutrition transition [14,15], most commonly consumed foods [31,36,55], and the dietary patterns reported by Faber et al. [51] for South African children. PC analysis for dietary pattern identification does not come without limitations, including subjective decisions on how to interpret and name patterns, the number of components to retain, and the threshold for factor loadings to be used in naming patterns [65]. The retained patterns typically also explain less than 50% of the variance explained. The five patterns in each age group in the present study explained almost a third of the variance, which compares well with the variance explained by the two patterns per age group by Faber et al. [51] (just more than a third). Principal factor analysis aims to maximize the fraction of variance explained by a weight linear combination of original variables, which does not necessarily increase the ability to discriminate between subjects with disease (malnutrition) or not. The present study’s data were self-reported and a cross-sectional design was used; hence, no causal links can be implied.

## 5. Conclusions

Bearing in mind the limitations of this study, we conclude that the dietary patterns in 1–<10-year-old children in the Western Cape contain strong elements of energy-dense, nutrient-poor Western dietary patterns, as at least one such a pattern was included in the top three strongest patterns in all three age groups that were investigated. Few of the dietary patterns included vegetables other than starchy vegetables, or fruit, dairy, quality proteins, and unrefined carbohydrates. Key predictors of greater adherence to the mostly unhealthy patterns included indicators of a higher socio-economic status in all three age groups, as well as having an obese mother in the 6–12-year-old group. As dietary habits and patterns formed in childhood continue into adulthood [10], the findings of this research point to an urgent need for review of the effectiveness of current policy and interventions aimed at ensuring child food security and well-being, as well as a review of policy and legislation aimed at supporting a healthy food environment, to identify drivers of the nutrition transition that may need further actioning to improve the dietary patterns of children in the country.

Key predictors of greater adherence to the mostly healthy patterns were a higher wealth index and having an obese mother in the two younger groups, with no predictors in the 6–<10-year-old group. There were no associations between any of the dietary patterns and stunting or overweight/obesity in the children. We recommend that interventions to improve the dietary intake of children should be directed at both poorer and higher income communities.

We foresee that the methodology for extraction of dietary patterns from dietary datasets that we established and described in detail in this study will be of value to others in the same field of research. The dietary patterns and socio-demographic predictors for 1–<10-year-olds we reported in this paper may inform the need for, and design of, further research for the monitoring of dietary patterns of South African children.

## Figures and Tables

**Table 1:** Food parameters and allocated foods for dietary pattern analyses.

Food Parameters	Terminology in Result Tables	Allocated Foods
Infant food	Infant food	Breast milk, breast milk substitutes, infant cereals
White bread	Bread white	White bread or rolls
Brown bread	Bread brown	Brown and whole wheat bread or rolls
Unrefined cereals	UCs	Hi-fibre breakfast cereals, e.g., All-Bran, Weetbix
Refined cereals	RCs	Refined breakfast cereals, sweetened and unsweetened
Maize porridge	Maize pap	Soft, stiff, and crumbly
Other refined carbohydrates	Rcarb-other	Rice, pasta, samp, mabella, mageu
Cheese	Cheese	Cheddar, gouda
Dairy	Dairy	Milk, yoghurt, and maas (sour milk)
Poultry	Poultry	With or without skin, any preparation
Red meat	Red meat	Beef, mutton, lamb and organ meat, any preparation
Processed meat	Proc meat	Cold meats, sausages, canned meat, dried meat
Eggs	Eggs	Any preparation
Fish	Fish	Fresh, canned, any preparation
Legumes	Legumes	Beans, lentils—soup and other preparations, soy mince
Vegetables: starchy	Veg-starchy	Potatoes, sweet potato, corn, sweet corn
Vegetables: starchy + fat	Veg-starchy + fat	“Slap chips” ^1^, potato roasted in fat, candied sweet potato
Vegetables: non starchy	Veg-non starchy	All vegetables except for starchy vegetables
Fruit	Fruit	Any fresh, canned or dried fruit, juice
Fats and oils: saturated	Fats-oils-sat	Butter, lard, hard margarine, coconut oil, non-dairy creamer
Fats and oils: unsaturated	Fats-oils-unsat	Soft margarine, plant oils, avocado, nuts, salad dressing
Refined carbohydrate + fat	Rcarb + fat	Savoury snacks—crisps, crackers
Refined carbohydrate + fat + sugar	Rcarb + fat + sugar	Cake, tarts, doughnuts, ice-cream, chocolates
Refined carbohydrate +protein + fat	Rcarb + prot + fat	Samoosas, pies, vetkoek, pizza, pasta dishes, fish cake
Refined carbohydrates + sugar	Rcarb + sug	Sweets: boiled, jelly-like
Sugar-sweetened beverages	SSBs	Fizzy drinks, squash, sport drinks
Sugar or syrup	Sugar	Granulated sugar, syrup, jam
Tea-coffee	Tea-coffee	Rooibos tea, Ceylon tea, coffee (no milk/sugar added)
Soup-sauces	Soup-sauces	Commercial soups, tomato sauce, chutney
Miscellaneous	Misc.	Condiments, Marmite, Bovril, fish paste

Mageu = fermented maize drink; Maas = sour milk; Vetkoek = balls of dough fried in oil; ^1^ French fries.

**Table 2:** Sociodemographic and other characteristics of the 1–<10-year-old children in the two provinces studied.

	Gauteng N = 733 % (95% CI)	Western Cape N = 593 % (95% CI)	Rao–Scott Chi-Sq Values	All N = 1326 % (95% CI)
Primary caregiver				
Mother	70.1 (65.6–74.6)	71.0 (64.7–77.2)	0.045 *	70.4 (66.8–74.0)
Father	6.6 (3.4–9.7)	1.8 (0.2–3.3)		5.0 (2.8–7.1)
Grandparent	16.7 (12.9–20.4)	21.0 (15.5–26.4)		18.1 (15.0–21.2)
Other (e.g., sibling, aunt)	6.7 (4.0–9.5)	6.3 (2.1–10.4)		6.6 (4.3–8.8)
Age in years				
1–<3 years	26.3 (22.1–30.6)	25.3 (19.4–31.2)	0.923	26.0 (22.6–29.4)
3–<6 years	35.4 (31.0–39.8)	35.1 (30.7–39.5)		35.3 (32.1–38.5)
6–<10 years	38.3 (34.1–42.4)	39.6 (33.1–46.1)		38.7 (35.2–42.2)
Gender				
Male	50.2 (45.5–54.9)	47.5 (43.1–51.9)	0.391	49.3 (45.9–52.7)
Female	49.8 (45.1–54.5)	52.5 (48.1–56.9)		50.7 (47.3–54.1)
Head of household				
Father	40.2 (33.8–46.6)	38.8 (34.6–43.0)	0.132	39.7 (35.3–44.1)
Mother	16.8 (13.8–19.9)	10.8 (7.0–14.5)		14.8 (12.5–17.2)
Grandmother	21.9 (15.5–28.3)	28.3 (21.8–34.9)		24.0 (19.3–28.8)
Grandfather	11.7 (8.3–15.1)	14.0 (10.0–18.0)		12.5 (9.9–15.0)
Other (e.g., aunt, uncle)	9.4 (5.7–13.1)	8.1 (4.9–11.4)		9.0 (6.3–11.7)
Marital status of mother				
Unmarried	41.1 (34.9–47.2)	34.8 (28.4–41.1)	<0.001 ***	39.0 (34.4–43.5)
Married	24.9 (20.5–29.4)	41.3 (33.3–49.2)		30.4 (26.4–34.3)
Divorced/widowed	4.8 (2.5–7.0)	2.4 (0.7–4.2)		4.0 (2.4–5.6)
Living together	27.8 (22.0–33.6)	20.8 (15.9–25.7)		25.5 (21.4–29.6)
Other	1.4 (0.2–2.6)	0.8 (0.0–1.8)		1.2 (0.3–2.1)
Mother’s highest education				
Not completing Gr. 12	51.2 (44.9–57.4)	57.7 (47.1–68.3)	0.183	53.3 (47.9–58.7)
Completion of Gr. 12	33.9 (28.4–39.4)	24.7 (17.6–31.8)		30.8 (26.5–35.2)
Qualification after Gr.12	12.2 (8.7–15.7)	15.6 (7.6–23.6)		13.3 (9.9–16.8)
Do not know	2.8 (1.4–4.1)	2.0 (0.5–3.5)		2.5 (1.5–3.5)
Father’s highest education				
Not completing Gr. 12	26.9 (22.0–31.7)	33.8 (29.0–38.5)	0.323	29.1 (25.6–32.7)
Completion of Gr. 12	32.6 (26.9–38.3)	30.4 (25.2–35.6)		31.9 (27.8–36.0)
Qualification after Gr.12	13.1 (9.4–16.9)	10.7 (5.7–15.7)		12.3 (9.4–15.3)
Do not know	27.4 (22.4–32.4)	25.2 (19.7–30.6)		26.7 (22.9–30.4)
Mother’s employment status				
Yes	22.4 (17.8–26.9)	38.4 (31.0–45.9)	<0.001 **	27.7 (23.9–31.5)
No	74.6 (69.6–79.6)	60.2 (53.0–67.5)		69.8 (65.8–73.9)
Do not know/not applicable	3.0 (1.3–4.7)	1.3 (0.3–2.4)		2.5 (1.3–3.6)
Father’s employment status				
Yes	64.8 (60.6–69.1)	65.3 (59.7–70.9)	0.953	65.0 (61.6–68.4)
No	21.4 (17.5–25.3)	20.5 (15.1–25.9)		21.1 (18.0–24.2)
Do not know/not applicable	13.8 (11.1–16.4)	14.1 (10.2–18.1)		13.9 (11.7–16.1)
Wealth index quintiles				
One	21.1 (14.6–27.6)	17.7 (10.7–24.7)	0.263	20.0 (15.1–24.8)
Two	17.8 (12.0–23.6)	24.3 (20.0–28.6)		20.0 (15.9–24.0)
Three	21.3 (17.0–25.7)	17.0 (12.6–21.4)		19.9 (16.7–23.1)
Four	21.5 (16.7–26.3)	17.5 (12.4–22.6)		20.2 (16.6–23.7)
Five	18.3 (11.6–25.0)	23.5 (14.5–32.5)		20.0 (14.7–25.3)
Ethnicity				
Black African	97.8 (96.0–99.6)	27.6 (12.9–42.3)	<0.001 **	74.5 (69.5–79.4)
Mixed ancestry	2.2 (0.3–4.0)	68.0 (53.7–82.4)		24.1 (19.2–28.9)
Other	0.0 (0.0–0.1)	4.4 (0.6–8.2)		1.5 (0.3–2.7)
Type of residence				
Rural	2.4 (0.7–4.1)	6.6 (1.6–11.5)	0.194	3.8 (1.9–5.7)
Urban formal	88.9 (82.3–95.4)	86.8 (79.1–94.5)		88.2 (83.2–93.2)
Urban informal	8.7 (2.7–14.7)	6.6 (1.7–11.5)		8.0 (3.7–12.3)
Mother’s BMI [39]				
Underweight/normal BMI = <18.5 and 18.5–24.9 kgm^2^	33.3 (28.0–38.5)	29.1 (23.6–34.5)	0.002 **	32.0 (28.0–35.9)
Overweight BMI = 25–29.9 kgm^2^	27.7 (23.6–31.8)	20.4 (16.5–24.3)		25.4 (22.4–28.5)
Obese BMI ≥ 30 kgm^2^	39.1 (35.8–42.3)	50.6 (43.0–58.1)		42.6 (39.4–45.8)
Hunger scale [25]				
Total score = 0: No risk	57.9 (49.5–66.3)	48.8 (38.9–58.7)	0.1483	54.9 (48.5–61.3)
1–4: At risk of hunger	22.1 (17.2–27.0)	28.9 (23.0–34.9)		24.4 (20.6–28.2)
5–8: Food shortage in house	20.0 (14.8–25.1)	22.3 (16.5–28.0)		20.7 (16.8–24.6)

95% CI, 95% confidence intervals; * Significant relationship between the variable and province, chi-squared *p*-value < 0.05; ** *p* < 0.01; *** *p* < 0.001; N-values reflect actual number of cases, estimates are adjusted using relevant weighting.

**Table 3:** Dietary patterns and significant socio-demographic predictors for 1–<3-year-old children in the Western Cape and Gauteng (*n* = 333).

**Pap Soup/Sauce Pattern**		**Tea/Coffee & Sugar Pattern**		**Mostly Unhealthy Snack Pattern**		**White Bread & Topping Pattern**		**Healthy Pattern**	
**Food Parameters**	**PL**	**Food Parameters**	**PL**	**Food Parameters**	**PL**	**Food Parameters**	**PL**	**Food Parameters**	**PL**
Maize pap	0.84	Tea and/or coffee	0.74	RC-Fat	0.52	Bread White	0.65	Fats-oils-Unsat	0.60
Soup-sauces	0.44	Sugar	0.72	RC-Fat-sugar	0.50	Processed meat	0.53	Veg non-starchy	0.41
Dairy	−0.39	Fats-oils-Sat	0.59	Bread Brown	0.42	Miscellaneous	0.36	Fish	0.31
RC-Other	−0.55	Legumes	0.33	SSB	0.41	Eggs	0.32	Poultry	−0.38
URC	−0.59			Fruit	0.36			RC-Sugar	−0.55
				Baby food	−0.52				
% Variance explained	2.16	% Variance explained	2.1	% Variance explained	2.0	% Variance explained	1.66	% Variance explained	1.6
**Pattern Predictors ^1^**	**PE (SE)** ***p*-Value ^2^**	**Pattern Predictors ^1^**	**PE (SE)** ***p*-Value ^2^**	**Pattern Predictors ^1^**	**PE (SE)** ***p*-Value ^2^**	**Pattern Predictors ^1^**	**PE (SE)** ***p*-Value ^2^**	**Pattern Predictors ^1^**	**PE (SE)** ***p*-Value ^2^**
HHH Other *-lesser adherence*	−0.29 (0.13) 0.034	Higher WI *-greater adherence*	0.04 (0.01) 0.013	PCG: Grandmother -*lesser adherence*	−0.38 (0.15) 0.015	Gauteng -*lesser adherence*	−0.27 (0.11) 0.015	HHH: Grandparent -*lesser adherence*	−0.29 (0.11) 0.009
Mother has Gr 12 -*lesser adherence*	−0.31 (0.09) <0.001	PCG: Other *-lesser adherence*	−0.4 (0.19) 0.035	Mother has Gr 12 *-greater adherence*	0.37 (0.12) 0.002	Mother obese -*lesser adherence*	−0.24 (0.11) 0.026	Gauteng *-greater adherence*	0.45 (0.11) <0.001
Father has Gr12+ -*less adherence*	−0.27 (0.14) 0.049	Hunger risk *-greater adherence*	0.35 (0.11) 0.002	Father has Gr12+ - *greater adherence*	0.4 (0.18) 0.028	PCG: Other -*lesser adherence*	−0.35 (0.18) 0.06	Mother overweight *-greater adherence*	0.31 (0.14) 0.023
Higher WI *-lesser adherence*	−0.03 (0.01) 0.016							Mother obese *-greater adherence*	0.33 (0.12) 0.007
Gauteng *-greater adherence*	1.23 (0.09) <0.001							Greater WI *-greater adherence*	0.03 (0.02) 0.04
Hunger risk *-greater adherence*	0.25 (0.1) 0.009								
Hunger present *-greater adherence*	0.33 (0.12) 0.008								

PL = pattern loading; RC = refined carbohydrates; sat = saturated; unsat = unsaturated; PE = parameter estimate; SE = standard error; WI = wealth index, PCG = Primary caregiver; PCG Other = sibling or aunt; HHH = head of household; HHH Other = aunt or uncle; Gr = grade; URCs = unrefined breakfast cereals, RC-Other = other refined carbohydrates, e.g., pasta, rice, and samp; RC-Fat-sugar = combination of refined carbohydrates, fat, and sugar, e.g., cake, tarts, doughnuts, ice cream, chocolates; RC-Fat = combination of refined carbohydrates and fat, e.g., crisps (any type) and salty biscuits; Miscellaneous = salty spreads and condiments; Soup-sauces = commercial powdered soup, tomato sauce, and chutney; RC- Sugar = sugar in the form of sweets; Veg non-starchy = all vegetables excluding starchy vegetables; Fats-oils-Sat = Butter, lard, hard margarine, coconut oil, non-dairy creamer; Fats-oils-Unsat = Soft margarine, plant oils, avocado, nuts, salad dressing; SSBs = sugar-sweetened beverages, e.g., fizzy drinks, squash, and sport drinks. ^1^ A positive parameter estimate indicates greater adherence and a negative parameter estimate lesser adherence to a dietary pattern. ^2^ Multiple regression model with backward elimination constructed for each pattern; only significant predictors that remained in the final model are included in the table. Dietary pattern scores were standardized with means of 0 and a unit standard deviation.

**Table 4:** Dietary patterns and socio-demographic predictors for 3–<6-year-old children in the Western Cape and Gauteng (*n* = 514).

**Tea/Coffee, Sugar, & Sandwich Pattern**		**Unhealthy Food & Snack Pattern**		**Starch & Poultry Pattern**		**Breakfast Item Pattern**		**Vegetable & Legume Pattern**	
**Food Parameters**	**PL**	**Food Parameters**	**PL**	**Food Parameters**	**PL**	**Food Parameters**	**PL**	**Food Parameters**	**PL**
Tea/coffee	0.85	Bread-White	0.65	RC-Other	0.71	Dairy	0.62	Legumes	0.41
Sugar-syrup	0.82	Veg-Starchy-F	0.55	Veg starchy	0.48	Fruit	0.57	Veg non-starchy	0.41
Fats-oils-Unsat	0.49	RC- Prot-Fat	0.41	Poultry	0.43	Cheese	0.46	Miscellaneous	0.40
Bread Brown	0.33	RC-Fat-sugar	0.41	Maize pap	−0.53	RC-Fort-Cereal	0.46	URC	−0.62
Fats-oils-Sat	0.31	Processed meat	0.36						
% Variance explained	2.2	% Variance explained	1.81	% Variance explained	1.74	% Variance explained	1.72	% Variance explained	1.68
**Pattern Predictors ^1^**	**PE (SE)** ***p*-Value ^2^**	**Pattern Predictors ^1^**	**PE (SE)** ***p*-Value ^2^**	**Pattern Predictors ^1^**	**PE (SE)** ***p*-Value ^2^**	**Pattern Predictors ^1^**	**PE (SE** ***p*-Value ^2^**	**Pattern Predictors ^1^**	**PE (SE)** ***p*-Value ^2^**
None		HHH: Grandparent *-greater adherence*	0.27 (0.1) 0.008	PCG: Other *-lesser adherence*	−0.37 (0.14) 0.009	PCG: Grandmother *-lesser adherence*	−0.32 (0.12) 0.008	Gauteng *-greater adherence*	0.66 (0.1) <0.001
		Gauteng *-lesser adherence*	−0.63 (0.09) <0.001	Gauteng *-lesser adherence*	−0.74 (0.09) <0.001	Gender: Female *-greater adherence*	0.24 (0.09) 0.006	Hunger present *-greater adherence*	0.25 (0.11) 0.022
		Greater WI *-greater adherence*	0.03 (0.01) 0.016	Mother overweight *-lesser adherence*	−0.30 (0.09) 0.001	Mother has Gr12+ *-greater adherence*	0.28 (0.14) 0.045		
		Mother employed *-greater adherence*	0.22 (0.1) 0.026			Father has Gr12 *-greater adherence*	0.37 (0.1) <0.001		
						Father has Gr12+ *-greater adherence*	0.42 (0.15) 0.006		
						Mother employed *-greater adherence*	0.41 (0.1) <0.001		
						Father employed *-lesser adherence*	−0.25 (0.1) 0.009		
						Greater WI *-greater adherence*	0.05 (0.01) <0.001		
						Gauteng *-lesser adherence*	−0.24 (0.1) 0.012		
						Mother obese *-greater adherence*	0.25 (0.09) 0.005		

PL = pattern loading; RCs = refined carbohydrates; URCs = unrefined carbohydrates; sat = saturated; unsat = unsaturated; PE = parameter estimate; SE = standard error; WI = wealth index, Gr = grade, PCG = Primary caregiver; PCG Other = sibling or aunt; HHH = head of household; HHH Other = aunt or uncle; RC-Other = other refined carbohydrates, e.g., pasta and rice; RC-Fat-sugar = combination of refined carbohydrates, fat, and sugar, e.g., cake, tarts, doughnuts, ice cream, chocolates; RC-Protein-Fat = combination of refined carbohydrates, fat and animal protein, e.g., samosa, fat cakes, pie, pizza, and lasagna pasta dishes; Miscellaneous = salty spreads and condiments; RC Sugar = sugar in the form of sweets; Veg non-starchy = all vegetables excluding starchy vegetables; Veg-starchy-F = starchy vegetables combined with fat, e.g., “slap chips” (French fries); Fats-oils-Sat = Butter, lard, hard margarine, coconut oil, non-dairy creamer; Fats-Oils-Unsat = Soft margarine, plant oils, avocado, nuts, salad dressing; SSB = sugar-sweetened beverages, e.g., fizzy drinks, squash, and sport drinks. ^1^ A positive parameter estimate indicates greater adherence and a negative parameter estimate lesser adherence to a dietary pattern; ^2^ Multiple regression model with backward elimination constructed for each pattern; only significant predictors remaining in the final model are included in the table. Dietary pattern scores were standardized with means of 0 and a unit standard deviation.

**Table 5:** Dietary patterns and socio-demographic predictors for 6–<10-year-old children in the Western Cape and Gauteng (*n* = 479).

**Mostly Unhealthy Pattern 1**		**Tea/Coffee, Sugar, & Milk Pattern**		**Mostly Unhealthy Pattern 2**		**White Bread & Topping Pattern**		**Starchy Pattern**	
**Food Parameters**	**PL**	**Food Parameters**	**PL**	**Food Parameters**	**PL**	**Food Parameters**	**PL**	**Food Parameters**	**PL**
RC-Fat	0.47	Sugar	0.85	RC-BF cereal	0.53	Bread White	0.84	RC-Other	0.68
SSB	0.44	Tea or coffee	0.82	Red meat	0.44	Fats-oils-UnSat	0.48	Veg starchy	0.46
Fruit	0.41	Dairy	0.56	RC-Prot-Fat	0.34	Processed meat	0.42		
URC	0.40			RC-Fat-Sugar	0.32	Bread Brown	−0.51		
RC Sugar	0.36			Veg non-starchy	−0.32				
Fish	−0.33			Fats oils Sat	−0.33				
Legumes	−0.39			Poultry	−0.46				
Maize pap	−0.50								
Variance explained	2.2%	Variance explained	2.1%	Variance explained	1.83%	Variance explained	1.67%	Variance explained	1.61%
**Pattern Predictors ^1^**	**PE (SE)** ***p*-Value ^2^**	**Pattern Predictors ^1^**	**PE (SE)** ***p*-Value ^2^**	**Pattern Predictors ^1^**	**PE (SE)** ***p*-Value ^2^**	**Pattern Predictors ^1^**	**PE(SE)** ***p*-Value ^2^**	**Pattern predictors ^1^**	**PE(SE)** ***p*-Value ^2^**
Father has Gr12 *-greater adherence*	0.19(0.09) 0.040	HHH: Mother *-less adherence*	−0.41(0.11) <0.001	Higher WI *-greater adherence*	0.05(0.01) <0.001	Gauteng *-less adherence*	−0.32(0.09) <0.001	HHH: Other *-greater adherence*	0.35(0.16) 0.3
Higher WI *-greater adherence*	0.03(0.01) 0.017	PCG: Other *-less adherence*	−0.28(0.12) 0.022	Hunger present *-less adherence*	−0.34(0.1) <0.001			PCG: Grandmother *-greater adherence*	0.41(0.11) <0.001
Gauteng *-less adherence*	−0.43(0.09) <0.001	Female *-less adherence*	−0.28(0.09) 0.001	Gauteng *-less adherence*	−0.34(0.09) <0.001			Father employed *-less adherence*	−0.25(0.09) 0.006
Mother obese *-greater adherence*	0.21(0.09) 0.018	Gauteng *-less adherence*	−0.34(0.09) <0.001	Mother has Gr12+ *-greater adherence*	0.21(0.13) 0.1			Gauteng *-less adherence*	−0.91(0.09) <0.001
Hunger risk *-less adherence*	−0.33(0.11) 0.002							Hunger risk *-greater adherence*	0.35(0.11) 0.001
Hunger present *-less adherence*	−0.7(0.11) <0.001							Hunger present *-greater adherence*	0.28(0.11) 0.01

PL = pattern loading; RCs = refined carbohydrates; sat = saturated; unsat = unsaturated; PE = parameter estimate; SE = standard error; WI = wealth index, PCG = Primary caregiver; PCG Other = sibling or aunt; HHH = head of household; HHH Other = aunt or uncle; Gr = grade; URCs = unrefined breakfast cereals; RC-Other = other refined carbohydrates, e.g., pasta, rice, and samp; RC-Fat = combination of refined carbohydrates and fat, e.g., crisps (any type) and salty biscuits; RC-Fat -sugar = combination of refined carbohydrates, fat, and sugar, e.g., cake, tarts, doughnuts, ice cream, chocolates; RC-Protein-Fat = combination of refined carbohydrates, fat, and animal protein, e.g., samosa, fat cakes, pie, pizza, and lasagna pasta dishes; RC Sugar = sugar in the form of sweets; Veg non-starchy = all vegetables excluding starchy vegetables; Fats-oils-Sat = Butter, lard, hard margarine, coconut oil, non-dairy creamer; Fats-Oils-Unsat = Soft margarine, plant oils, avocado, nuts, salad dressing; SSBs = sugar-sweetened beverages, e.g., fizzy drinks, squash, and sport drinks. ^1^ A positive parameter estimate indicates greater adherence, and a negative parameter estimate indicates lesser adherence to a dietary pattern. ^2^ Multiple regression model with backward elimination was constructed for each pattern; only significant predictors remaining in the final model are included in the table. Dietary pattern scores were standardized with means of 0 and a unit standard deviation.

## Data Availability

The data presented in this study are available on request from the corresponding author pending ethical approval from the Faculty of Health Sciences Human Research Ethics Committee, University of Cape Town.
